# Optimizing *Akkermansia muciniphila* Isolation and Cultivation: Insights into Gut Microbiota Composition and Potential Growth Promoters in a Chinese Cohort

**DOI:** 10.3390/microorganisms12050881

**Published:** 2024-04-28

**Authors:** Xiangyu Meng, Chen Xv, Jiaping Lv, Shuwen Zhang, Changlu Ma, Xiaoyang Pang

**Affiliations:** 1 Institute of Food Science and Technology, Chinese Academy of Agricultural Sciences, Beijing 100193, China; mlr0723@163.com (X.M.); xu123chen456@163.com (C.X.); lvjiapingcaas@126.com (J.L.); zhangshuwen@caas.cn (S.Z.); machanglu@126.com (C.M.); 2 College of Agronomy and Biotechnology, Southwest University, Chongqing 400715, China

**Keywords:** *Akkermansia muciniphila*, 16S rRNA, separation and identification, growth promoting factor, metagenomics

## Abstract

The study aims to analyze the composition of the gut microbiota in Chinese individuals using metagenomic sequencing technology, with a particular focus on the abundance of *Akkermansia muciniphila* (Akk). To improve the efficiency of Akk isolation and identification accuracy, modifications were made to the enrichment culture medium and 16S rRNA universal primers. Additionally, potential growth-promoting factors that stimulate Akk growth were explored through in vitro screening. The research results revealed that the abundance of Akk in Chinese fecal samples ranged from 0.004% to 0.4%. During optimization, a type of animal protein peptide significantly enhanced the enrichment efficiency of Akk, resulting in the isolation of three Akk strains from 14 fecal samples. Furthermore, 17 different growth-promoting factors were compared, and four factors, including galactose, sialic acid, lactose, and chitosan, were identified as significantly promoting Akk growth. Through orthogonal experiments, the optimal ratio of these four growth-promoting factors was determined to be 1:1:2:1. After adding 1.25% of this growth-promoting factor combination to the standard culture medium, Akk was cultivated at 37° for 36 h, achieving an OD_600nm_ value of 1.169, thus realizing efficient proliferation and optimized cultivation of Akk. This study provides important clues for a deeper understanding of the gut microbiota composition in Chinese individuals, while also offering effective methods for the isolation and cultivation of Akk, laying the groundwork for its functional and application research in the human body.

## 1. Introduction

*Akkermansia muciniphila* (abbreviated as Akk) is a bacterial species isolated from human feces in the Netherlands by researchers at Wageningen University in the early 21st century [1]. As a representative of the phylum *Verrucomicrobia*, Akk belongs to the genus *Akkermansia* and is characterized as an obligately anaerobic or microaerophilic Gram-negative bacterium. It exhibits non-motility and non-spore-forming properties, possessing a unicellular morphology with a length ranging from 0.6 to 1.0 μm and capable of solitary or paired growth. Among the diverse gut microbiota, Akk holds a unique ecological niche by utilizing mucin exclusively as its carbon and nitrogen source, providing it with a distinctive survival advantage [2].

In recent years, with the deepening focus on gastrointestinal health, Akk has garnered significant attention for its robust probiotic functions. Akk primarily resides in the gastrointestinal tract’s outer mucus layer, which is predominantly composed of water (90–95%), electrolytes, lipids (1–2%), and proteins, with mucin serving as the principal structural and functional component. This environment provides the necessary conditions for Akk’s growth. Unlike most potentially pathogenic mucin-degrading microbes, the presence of Akk is beneficial to the host, playing a crucial role in probiotic functions. Akk utilizes its endogenously encoded array of degradative enzymes [3], such as sialidase, fucosidase, and sulfatase, to break down mucin into metabolites like acetate, propionate, and 1,2-propanediol [4]. These metabolic activities are pivotal in modulating host immune responses and lipid metabolism [1,5,6,7]. Situated in the outer mucus layer without penetrating the inner layer, Akk contributes to maintaining gut barrier integrity by promoting the natural renewal of the outer mucus layer [8]. Additionally, Akk exhibits unique anti-inflammatory properties and immunomodulatory functions [9], evident in its ability to reduce pro-inflammatory factors’ expression and suppress the activity of pro-inflammatory immune cells in various mouse models. In the context of host metabolism, Akk can reverse metabolic dysregulations induced by high-fat diets, such as adipose tissue inflammation, insulin resistance, and endotoxemia [10,11,12,13]. In diabetes treatment, Akk has been found to synergize with metformin to improve blood sugar levels [14]. Moreover, research linking Akk to human health has uncovered its significant associations with conditions like alcoholic liver disease, cancer, and neurodegenerative diseases. Clearly, Akk occupies an important position in the realm of human health. An in-depth exploration of Akk will enhance our comprehensive understanding of the interactions between gut microbiota and host health, offering new perspectives and directions for improving human well-being.

Numerous factors influence the abundance of Akk within the host’s intestinal tract. Research indicates that Akk colonization in the gastrointestinal tract begins in infancy, with its abundance rapidly increasing within the first year to levels observed in adults, and subsequently declines with age [15]. Geographic variations also contribute to disparities in Akk abundance among different populations, with individuals in Southern China exhibiting generally lower levels compared to Europeans. Additionally, diet and lifestyle significantly impact Akk’s presence in the human body. Diets high in protein, fats, sugars, and saturated fats notably decrease Akk abundance in the gut [16,17,18], whereas ketogenic diets, foods rich in polyunsaturated fatty acids, and polyphenols promote its proliferation in the intestinal tract [19,20]. The use of medications, particularly antibiotics such as vancomycin, piperacillin, hydroxychloroquine, and imipenem, has been identified as a key factor affecting Akk levels. Studies by Dubourg et al. have shown that the administration of antibiotics can significantly increase Akk quantities in adult patients, while a combination of antibiotics (ampicillin, vancomycin, neomycin, and metronidazole) can markedly reverse the reduction in Akk numbers caused by immunosuppressants in mice [21]. Thus, dietary interventions offer a substantiated approach to modulating Akk abundance in the host’s gut [22,23].

Akk represents the sole known member of the *Verrucomicrobia* phylum within the human gut, encompassing various subspecies within the *Akkermansia* genus, including *Akkermansia muciniphila, Akkermansia biwaensis* and *Akkermansia glycaniphila*. However, the isolation and differentiation of Akk strains pose significant challenges, with the majority of research hitherto relying on the model strain A. muciniphila MucT (ATCC BAA-835) isolated by Wageningen University, alongside a limited number of other specific strains. Given Akk’s rich intraspecific diversity, the identification of novel Akk strains from the Chinese population is of paramount importance for research purposes [24]. The stringent anaerobic nature of Akk strains, their exacting extracorporeal growth requirements, and slow growth rates complicate their isolation, conferring a competitive disadvantage during the isolation process and thereby limiting successful isolation to a handful of laboratories [25]. To date, fewer than 40 Akk strains have been isolated from Chinese fecal samples, significantly impeding comprehensive studies on the gut Akk population in the Chinese demographic [19]. Moreover, the industrial application of Akk faces considerable challenges due to domestic legal and regulatory constraints, precluding the direct oral administration of live Akk cultures to promote host health. Consequently, indirect strategies to harness Akk’s probiotic functions may represent a breakthrough in its industrial application within China. Studies have demonstrated that various exogenous substances, including arabinoxylan, lentil husks, L-rhamnose, Pu-erh tea, Yukihikari rice, berberine, dietary inulin, cranberry extract, and raw potato starch, positively impact Akk abundance, indicating the feasibility of increasing gut Akk levels through external interventions [26,27,28,29,30,31]. Therefore, identifying growth-promoting factors for Akk and incorporating foods rich in these factors can enhance the endogenous Akk population in the host’s gut, leveraging its probiotic properties and offering a safer, innovative approach to modulating human physiological health through Akk regulation.

This study employed metagenomic sequencing technology to investigate the distribution of *Akkermansia muciniphila* within the gastrointestinal tract of the Chinese population. By refining the enrichment and isolation protocols for Akk, we conducted the isolation of Akk strains indigenous to China. The research focused on various growth-promoting factors, including polyphenols, oligosaccharides, and sialic acid, to carry out in vitro screening experiments. This process identified the most effective combination of growth-promoting factors, laying the groundwork for the industrial application of Akk in the Chinese demographic.

## 2. Materials and Methods

### 2.1. Materials

The BHI medium was purchased from Land Bridge Technology Co., Ltd. (Beijing, China). Chitosan, lactose, galactose, L-rhamnose, and kelp polysaccharide were purchased from Shanghai Yuanye Biotechnology Co., Ltd. (Shanghai, China). Yam peptide and casein peptide were obtained from the food laboratory of Institute of Agricultural Products Processing, Chinese Academy of Agricultural Sciences. Cranberry extract and fucose were purchased from Qingdao Youshun Genesis Technology Co., Ltd. Sialic acid was purchased from Aladdin Biochemical Technology Co., Ltd. (Shanghai, China). All solvents and of analytical were analytical grade and purchased from Sinopharm Chemical Reagent Co., Ltd. (Shanghai, China).

### 2.2. Samples and Media

A total of 14 fecal samples were collected from Chinese people aged 6–43 years. The information about the fecal samples is shown in Table 1.

Enriched medium: 1 g/L L-cysteine hydrochloride, 50 mL/L animal protein peptides, 0.2 g/L KH_2_PO_4_, 0.53 g/L Na_2_HPO_4_, 0.3 g/L NH_4_Cl, 4 g/L NaHCO_3_, 0.4g/L KH_2_PO_4_, 0.0005 g/L C_12_H_6_NNaO_4_.

Primary screening medium: 40 g/L BHI, 1 g/L L-cysteine hydrochloride, 20 g/L agar, 50 mL/L animal protein peptides.

Re-screening medium: 40 g/L BHI, 1 g/L L-cysteine hydrochloride.

### 2.3. Analysis of the Gut Microbiota in the Chinese Population

#### 2.3.1. Collection of Fecal Samples

Fecal samples were collected from a cohort of healthy individuals, employing sterile spoons to obtain interior portions of fresh stool specimens, carefully avoiding parts that had come into contact with air. The specimens were promptly placed into sterile, enzyme-free fecal collection tubes. Samples were aliquoted aseptically, with each aliquot weighing over 2 g, and subsequently transported to the laboratory for storage in a −80° ultra-low temperature freezer until further analysis.

#### 2.3.2. Metagenomic Sequencing Protocol

Fecal DNA was extracted using the Magnetic Soil and Stool DNA Kit (DP712) following the manufacturer’s instructions. DNA purity was assessed using a Nanodrop spectrophotometer. Approximately 50 ng of each sample was subjected to 1% agarose gel electrophoresis to evaluate the integrity of the extracted genomic DNA. Samples exhibiting a single, bright band without significant smearing were deemed satisfactory. The concentration of genomic DNA was precisely quantified using a Qubit fluorometer. The construction of genomic libraries and subsequent sequencing were conducted by GenePlus Medical Laboratory, employing the DNBseq sequencing platform for metagenomic analysis.

#### 2.3.3. Bioinformatics Analysis

Sequencing data quality control was performed using KneadData software (Version: 0.7.10). An initial quality assessment of raw sequencing data was conducted with FastQC software (Version: 0.11.9), followed by the trimming of adapters and low-quality bases, and filtration of low-quality sequences using Trimmomatic software (Version: 0.33) [32]. Host sequence alignment was executed using Bowtie2 (Version: 2.2.3) [33] to eliminate host sequence contamination from the sequencing data. Subsequent sequence quality verification was carried out using FastQC software to ensure that sequence quality met analysis standards. Quality metrics of raw sequencing data, including insert size, number of sequencing reads, base count, GC content, and percentages of bases with Phred scores exceeding 20 (Q20) and 30 (Q30), were compiled. Species annotation was performed using MetaPhlAn 3.0 software, with quality-controlled sequences aligned against a marker gene database using Bowtie2 (Version: 2.4.2) to rapidly ascertain the composition of the microbial community, including bacteria, archaea, eukaryotes, and viruses. Metagenomic data analysis was conducted by employing the bioBakery 3 [34] workflow to analyze microbial species abundance.

### 2.4. Isolation and Identification of Akk

#### 2.4.1. Preparation of Enzymatic Peptide Solutions

A solution containing 50 g of animal protein, at a substrate concentration of 5%, was incubated in a 40° water bath. The pH of the protein solution was adjusted to 7.2 and maintained under slow stirring until stabilization, followed by the addition of 1000 U/g of neutral protease for enzymatic digestion. During the initial 45 min of digestion, 3 mol/L NaOH was periodically added every 5 min to maintain a constant pH, followed by pH adjustments every 20 min until the completion of the reaction. The sample was then boiled in a water bath for 10 min, cooled to room temperature, and centrifuged at 8000 rpm for 20 min. The supernatant was stored at −20° for further use.

#### 2.4.2. Isolation and Identification of Akk

The isolation of Akk was conducted following the experimental methodology outlined by Feng et al. [35]. Fecal samples were serially diluted and inoculated into enrichment culture media, followed by incubation at 37° in an anaerobic chamber for 72 h. Bacterial genomic DNA was extracted using a Bacterial Genome DNA Extraction Kit (DP302-02) according to the manufacturer’s instructions. Specific primers (AM1: 5′-CAGACGTGAAGGTGGGGAC-3′; AM2: 5′-CCTTGCGGTTGGCTTCAGAT-3′) were used for Polymerase Chain Reaction (PCR) amplification. The reaction mix included 2 × PCR Master Mix 12.5 μL, AM1 1 μL, AM2 1 μL, and template DNA 3 μL, supplemented with ddH2O up to 25 μL. Amplification conditions were as follows: initial denaturation at 95° for 5 min, followed by 30 cycles of denaturation at 95° for 30 s, annealing at 60° for 30 s, and extension at 72° for 1 min, with a final extension at 72° for 10 min. PCR products were validated via 1% agarose gel electrophoresis. Tubes showing a 327 bp band were diluted and plated onto a primary screening solid medium and incubated anaerobically at 37° for 2 weeks. Suspected colonies were subcultured, and the DNA of suspected bacteria was extracted for 16S rRNA gene testing. The universal primers 27F and 1492R were optimized for the Akk 16S sequencing region, using modified primers (27F-NM: 5′-AGAGTTTGATNMTGGCTCAG-3′; 1492R: 5′-TACGGYTACCTTGTTACGACTT-3′) for amplification. The 16S amplification products were sequenced by GeneTech Biotechnology Co., Ltd. (Beijing, China). Sequencing results were assembled using MEGA software(Version: 11), and the complete strain base sequences were submitted to the National Center for Biotechnology (NCBI) database for BLAST sequence comparison. A phylogenetic tree was constructed in conjunction with the sequences of the most similar type strains.

### 2.5. In Vitro Screening of Akk Growth Promoting Factors

#### 2.5.1. Single-Factor Experiments for In Vitro Screening

Seventeen growth-promoting factors including trehalose, kelp polysaccharides, L-rhamnose, fructose, maltose, lactose, beet extract, psyllium husk powder, cranberry extract, sialic acid, fucoidan, sorbitol, yam peptides, galactose, oligogalactose, chitosan, and casein peptides were added separately to BHI culture media containing 0.25% animal protein peptides to screen for growth-promoting factors. Akk activated for two generations was inoculated at a 4% inoculation rate into BHI culture media supplemented with different growth-promoting factors, ensuring anaerobic culture for 60 h. The OD_600nm_ values were measured using a multifunctional microplate reader. The growth-promoting factors that showed the best effect on Akk growth were selected for subsequent experiments.

#### 2.5.2. Orthogonal Experiments for In Vitro Screening

Based on the results of the single-factor screening experiments and using OD_600nm_ values as the evaluation indicator, L-rhamnose, sialic acid, galactose, and chitosan were selected as significant factors influencing Akk growth. A L_9_(3^4^) orthogonal experiment design was implemented with three levels set at 0.25%, 0.5%, and 1% on a BHI culture medium base of 40 g/L, supplemented with 1 g/L L-cysteine hydrochloride and 0.25% enzymatic animal protein peptides.

### 2.6. Data Processing

Graphs and charts were analyzed and generated using GraphPad Prism 8.0.

## 3. Results and Discussion

### 3.1. Metagenomic Sequencing Data Statistics

The sequencing data were subjected to quality control and filtering, and a total of 1,343,073,984 valid reads were obtained from 14 feces of adult healthy individuals. Q20 and Q30 reached over 96% and 94%, respectively, indicating high reliability of the sequencing data (Table 2).

### 3.2. Composition of Gut Microbiota in Chinese Population

The intricate relationship between gut microbiota and host health underscores the significance of analyzing the gut microbial composition of the Chinese population. Such analyses are instrumental not only in gaining a deeper understanding of the gut health of Chinese individuals but also in providing a crucial foundation for examining the prevalence of Akk within the Chinese gut microbiome. Accordingly, this study employed metagenomic sequencing technology to assess the microbial composition within the guts of Chinese individuals across various age groups.

**Figure 1:**
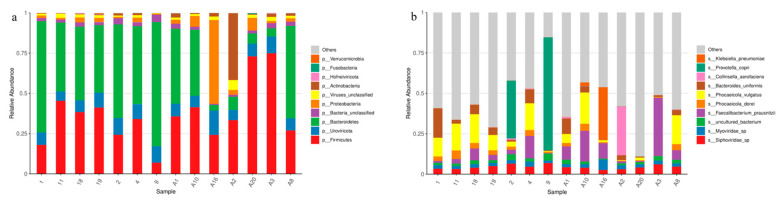
Relative abundance of intestinal flora. (**a**) The relative abundance of 14 Chinese gut microbiota at phylum. levels. (**b**) The relative abundance of 14 Chinese gut microbiota at species levels.

**Figure 2:**
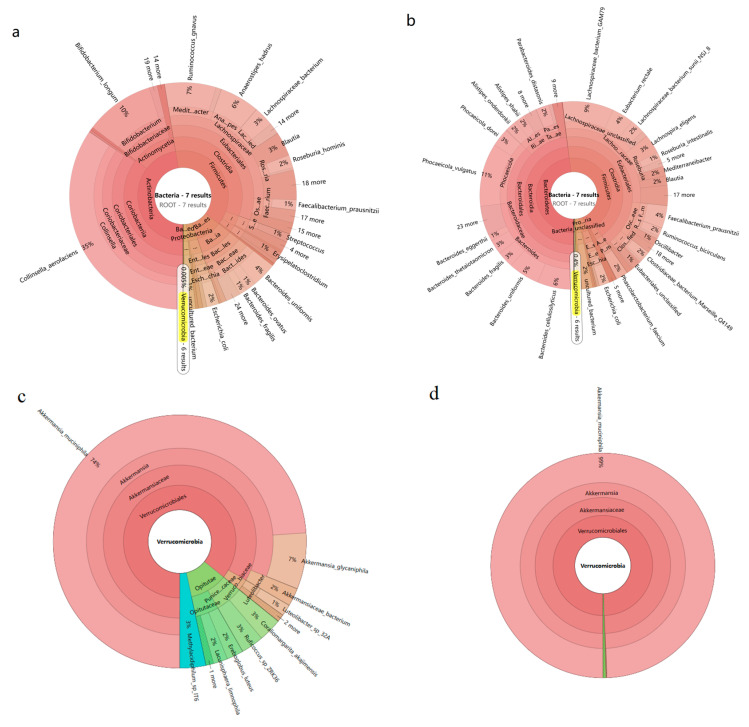
The Krona map was analyzed by metagenomic sequencing technology. (**a**) Abundance of *Verrucomicrobia* in sample A2. (**b**) Abundance of *Verrucomicrobia* in sample 19. (**c**) Abundance of *Akkermansia muciniphila* in sample A2. (**d**) Abundance of *Akkermansia muciniphila* in sample 19.

The findings from fourteen analyzed samples indicated that the predominant phyla within the gut microbiota were *Bacteroidetes*, accounting for 39.41 ± 24.37%, and *Firmicutes*, representing 36.96 ± 18.71%. Other phyla with relatively high abundance included *Proteobacteria* (6.26 ± 13.35%), *Actinobacteria* (3.83 ± 10.85%), and *Fusobacteria* (0.06 ± 0.13%) (Figure 1a). The most prevalent bacterial species identified were *Phocaeicola vulgatus* (8.69 ± 7.68%) and *Faecalibacterium prausnitzii* (8.03 ± 9.75%). Secondary abundances were noted for *Prevotella copri* (7.56 ± 20.3%), *Bacteroides uniformis* (4.48 ± 4.93%), *Klebsiella pneumoniae* (2.52 ± 8.71%), *Collinsella aerofaciens* (2.37 ± 8.02%), and *Phocaeicola dorei* (2.27 ± 1.66%) (Figure 1b). At the phylum level, *Verrucomicrobia* comprised 0.005–0.4% of the gut microbiota (Figure 2a,b), with the *Akkermansia* genus, including *Akkermansia muciniphila* and *Akkermansia glycaniphila*, being identified within this phylum. On a species level, the abundance of *Akkermansia muciniphila* exhibited significant variability across different age groups within the Chinese population, with abundances roughly ranging from 0.004% to 0.4% (Figure 2c,d). Although existing studies suggest Akk typically constitutes 0.5–5% of the total gut bacterial mass, the results of this investigation reveal a notably lower prevalence of Akk in the Chinese population across various age groups compared to data reported internationally, indicating a relatively lower abundance of Akk within the Chinese demographic.

### 3.3. Isolation and Screening of Akk Bacteria

Given the exceedingly low abundance of Akk in the gut and the absence of a specifically tailored isolation medium that selectively promotes its growth, the isolation of Akk has consistently presented significant challenges. In this study, an enriched culture medium was modified by the incorporation of enzymatically hydrolyzed animal protein peptides, facilitating the successful enrichment culture of 14 samples. Subsequently, positive enrichment culture tubes that were turbid and homogeneous at high dilution gradients were selected for subsequent isolation experiments, with the enrichment status of each sample detailed in Table 3. After the enrichment culture, a total of 99 turbid enrichment culture tubes were obtained, of which 45 were homogeneously turbid positive tubes. Positive enrichment culture tubes at higher dilution gradients were selected for PCR product electrophoresis, yielding 10 samples with a target band of 327 bp. Samples with clear and bright bands at high dilution gradients were further diluted and cultured in the initial screening medium.

**Figure 3:**
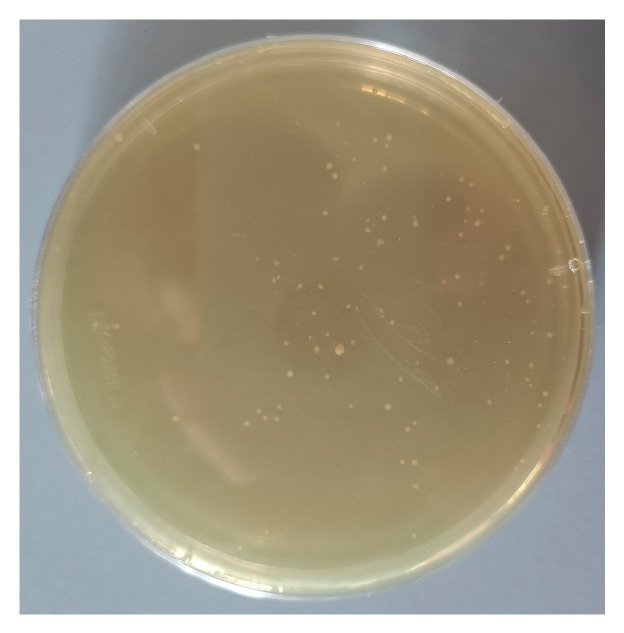
The Krona map was analyzed by metagenomic sequencing technology.

**Figure 4:**
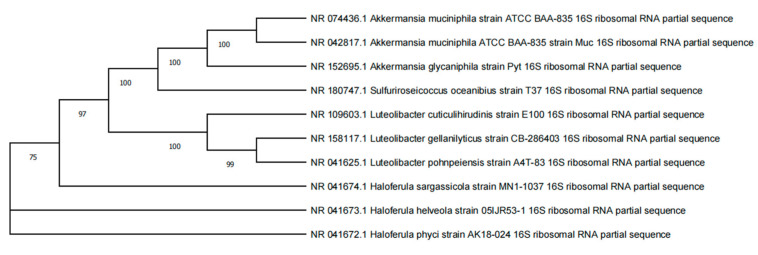
Phylogenetic tree based on 16S rRNA *Akkermansia muciniphila.*

Following the initial screening culture, suspect colonies were illustrated in Figure 3. Colonies that were semi-transparent, smaller than 1 mm in diameter, and possessed a viscous consistency were selected and cultured in a secondary screening medium. Gram staining and microscopy were performed on the bacterial fluid from positive secondary screening tubes, with PCR identification carried out on tubes that yielded Gram-negative results. The sequences of the strains obtained were submitted to the NCBI database for alignment. Using MEGA software, a phylogenetic tree was constructed based on nucleic acid sequences using the Neighbor-Joining (NJ) method after conducting a multiple sequence alignment of the top ten strains and normalizing the sequences by trimming divergent ends, as shown in Figure 4.

The final comparison revealed the successful isolation of three Akk strains from 14 samples. The results of the isolation and screening indicated that the medium supplemented with enzymatically hydrolyzed peptides had a 70% success rate in enrichment culture, and a 45% likelihood of presumptive positive bacteria in the initial screening medium, suggesting that the addition of animal protein peptides effectively enriched Akk and enhanced the efficiency of its isolation and screening. Moreover, the preparation method for the hydrolyzed peptide solution was straightforward, addressing the cumbersome and time-consuming issues associated with medium preparation, and resulting in a clearer medium that facilitated the observation of bacterial growth. Furthermore, during the identification process of Akk, it was discovered that the commonly used primer for 16S sequencing, 27F, could not successfully bind to the newly isolated Akk strains. Consequently, this study optimized the 27F primer based on the genomic sequences of Akk in the NCBI bacterial library, providing a more accurate method for the identification and subsequent quantification of Akk.

### 3.4. In Vitro Screening Results of Growth Promoting Factors

#### 3.4.1. Single-Factor Test Results

Akk as an emerging probiotic, has been the subject of considerable debate regarding its application in the food industry. A specialized medical food product containing the Akk strain WB-STR-0001 has been approved and launched in the United States, and Rob Knight has successfully secured funding to initiate research on the use of Akk for the treatment of Amyotrophic Lateral Sclerosis (ALS). These developments underscore the practical value of Akk. However, its direct application in foods is hindered by stringent domestic regulations and the challenges associated with high-density fermentation cultures. Given the safety of Akk has not been conclusively established, leveraging its probiotic effects indirectly through the addition of exogenous substances represents a particularly critical and viable strategy.

**Figure 5:**
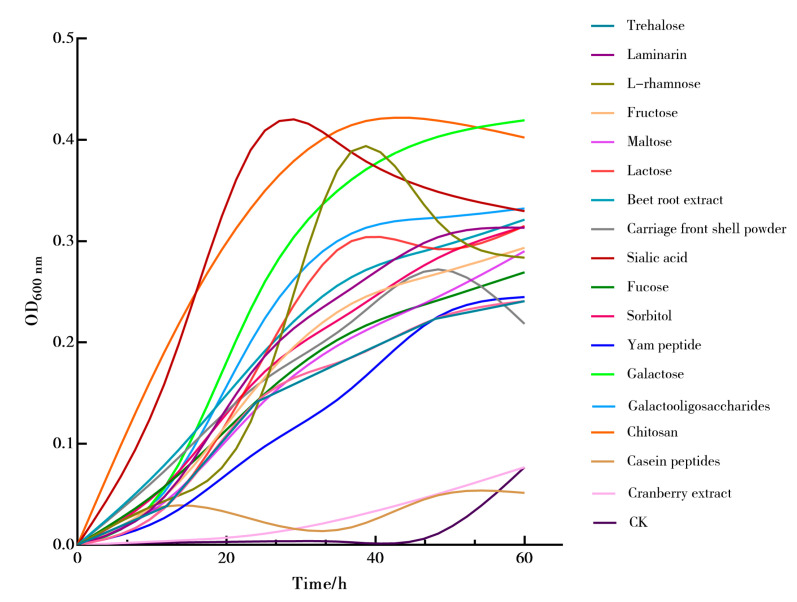
The growth curve of Akk among 17 growth factors.

Although previous research has mentioned various substances that promote the growth of Akk, there is a lack of systematic comparison and screening. Therefore, this study focused on the exploration of growth-promoting factors for Akk, evaluating 17 different substances including sugars, peptides, and plant extracts to identify those that effectively enhance Akk growth. Using a culture medium without growth-promoting factors as the control, different growth-promoting substances were added to brain heart infusion broth used for culturing Akk under anaerobic conditions, and their optical density at 600 nm (OD_600nm_) was measured. The aim was to indirectly increase its abundance in the gut. As illustrated in Figure 5, L-rhamnose, sialic acid, galactose, and chitosan showed the most significant promotion of Akk growth, with the maximum OD_600nm_ values of the Akk growth curves exceeding 0.35. Oligogalactose, lactose, and kelp polysaccharides exhibited secondary effects in promoting Akk growth.

#### 3.4.2. Orthogonal Test Results

**Figure 6:**
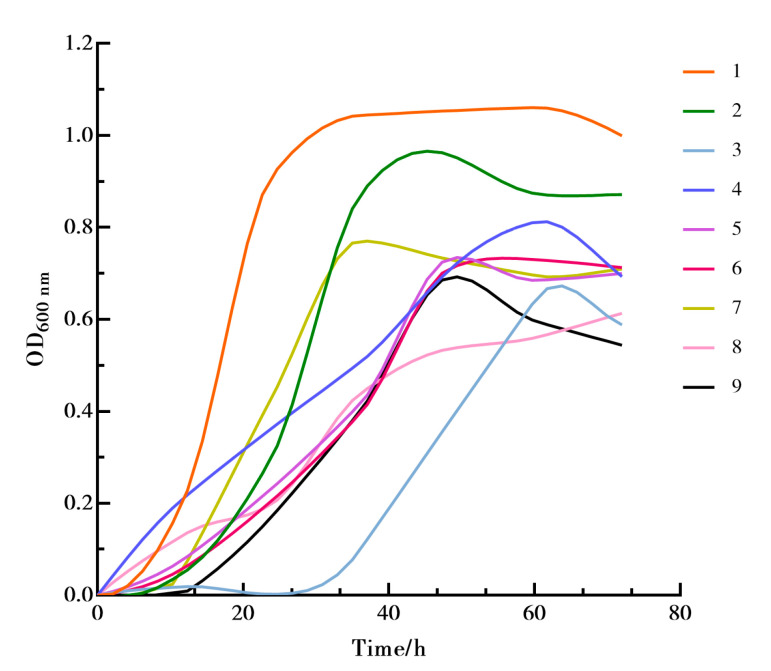
The growth curve of Akk under orthogonal test.

Through single-factor experiments, four growth-promoting factors were identified: L-rhamnose, sialic acid, galactose, and chitosan, set at three levels of concentration: 0.25%, 0.5%, and 1%. Activated bacterial cultures, in their second generation, were inoculated at a 4% inoculum volume into anaerobic culture media containing various combinations of these growth-promoting factors, corresponding to the orthogonal experimental design. Growth curves were plotted based on the OD_600nm_ values measured for each combination, as depicted in Figure 6. Variance analysis was conducted using the highest measured OD_600nm_ values to identify the combination of growth-promoting factors that yielded the best growth outcomes for Akk. As shown in Table 4, the optimal combination for enhancing Akk growth was identified as A_1_B_1_C_1_D_1_.

Visual analysis, as presented in Table 4, indicated that chitosan was the most significant factor, followed by L-rhamnose, with the order of influence on growth factors being A > B > D > C. The best combination for promoting Akk growth from a visual analysis perspective was A_1_B_1_C_2_D_1_. Validation experiments conducted at this level revealed the highest OD_600nm_ value for Akk growth to be 1.169, consistent with the results from the orthogonal experiment. Therefore, the findings from the orthogonal experiment suggest that the most effective promotion of Akk growth occurs when the ratio of the four growth-promoting factors—chitosan, L-rhamnose, sialic acid, and galactose—is set at 1:1:2:1.

## 4. Conclusions

In summary, the discovery of *Akkermansia muciniphila* has opened new avenues for the prevention of metabolic disease symptoms. With ongoing research into the mechanisms by which this bacterium influences metabolic diseases, coupled with an increased understanding and appreciation of the gut microbiota, Akk holds substantial potential for application in health foods.

This study utilized metagenomic sequencing technology to analyze the gut microbiota composition of the Chinese population, revealing that the *Verrucomicrobia* phylum constitutes 0.005–0.4% of the gut microbiota, with Akk’s abundance ranging only from 0.004% to 0.4%, indicating a relatively low prevalence. Furthermore, by optimizing culture mediums and refining 16S rRNA sequencing primers, the efficiency of Akk isolation and identification was enhanced, resulting in the successful isolation of three Akk strains from 14 samples. Additionally, single-factor experiments identified 17 potential growth-promoting factors for Akk among sugars, peptides, and plant extracts, with L-rhamnose, oligogalactose, sialic acid, and chitosan showing the most significant growth-promoting effects. These findings demonstrate that exogenous substances, through their unique bioactive properties, can act on Akk to promote its growth. Orthogonal experiments and the measurement of Akk growth curves were conducted to optimize the combination and concentration of these four growth-promoting factors, establishing the optimal ratio of chitosan: L-rhamnose: sialic acid: galactose as 1:1:2:1 with a concentration of 1.25%, thereby providing strong support for the efficient proliferation of Akk.

However, the specific mechanisms by which these substances promote the growth of Akk bacteria remain unclear. Future research should delve deeper into the molecular structures, targets, and signaling pathways of these substances to uncover the intrinsic mechanisms underlying their promotion of Akk growth.

## Figures and Tables

**Table 1:** Sample collection information.

Sample Number	Region	Age	Sexuality
19	Shanxi	28	Male
8	Shandong	25	Female
9	Inner Mongolia	40	Male
11	Hubei	35	Female
A2	Beijing	8	Female
18	Hebei	25	Male
3	Gansu	24	Female
16	Hubei	26	Female
10	Shandong	23	Female
A1	Beijing	5	Male
20	Hebei	22	Female
2	Chongqing	24	Female
1	Harbin	25	Female
4	Shandong	43	Male

**Table 2:** An amount of 14 Chinese fecal metagenomic sequencing data.

Sample	Original Reads	Filtering Reads	Final Reads	Q20/%	Q30/%	GC Content/%
19	80,585,330	76,475,373	75,983,124	96.59	95.57	46.80
8	105,779,054	101,703,871	101,044,837	98.79	96.06	44.89
9	83,067,148	80,090,271	79,199,880	98.39	95.62	45.84
11	124,866,176	121,205,833	120,750,312	98.62	95.95	42.90
A2	83,716,228	79,659,365	79,245,023	97.9	96.08	49.44
A1	105,779,054	93,292,019	90,780,817	97.50	95.31	52.54
3	100,350,480	99,974,632	99,964,808	98.34	94.79	49.36
16	83,048,708	82,698,892	81,715,347	98.46	95.23	50.23
10	87,282,956	86,986,876	86,978,522	98.19	94.26	46.36
18	105,047,450	101,093,277	100,721,568	99.00	96.76	45.08
20	88,333,690	87,959,542	87,922,092	98.22	94.43	44.75
2	129,656,978	125,185,953	124,092,457	98.26	95.17	45.97
1	115,531,846	111,946,287	111,485,261	98.16	95.52	46.52
4	99,367,970	94,801,793	94,384,979	98.29	95.21	46.82

**Table 3:** Turbidity of enrichment tube of 14 samples.

Sample	Dilution									
19	10^−2^ ① +	10^−2^ ② +	10^−3^ ① +	10^−3^ ② −	10^−3^ ③ +	10^−4^ ① +	10^−4^ ② +	10^−4^ ③ −	10^−5^ ① +	10^−5^ ② −
8	10^−1^ ① +	10^−2^ ② +	10^−3^ ① +	10^−3^ ② −	10^−3^ ③ +	10^−4^ ① +	10^−4^ ② +	10^−4^ ③ −	10^−5^ ① −	10^−5^ ② −
9	10^−2^ ① +	10^−2^ ② +	10^−3^ ① −	10^−3^ ② +	10^−3^ ③ +	10^−4^ ① +	10^−4^ ② −	10^−4^ ③ −	10^−5^ ① +	10^−5^ ② +
11	10^−1^ ① +	10^−2^ ② +	10^−3^ ① +	10^−3^ ② +	10^−3^ ③ +	10^−4^ ① +	10^−4^ ② −	10^−4^ ③ −	10^−5^ ① +	10^−5^ ② +
13	10^−1^ ① +	10^−2^ ② +	10^−3^ ① +	10^−3^ ② +	10^−3^ ③ +	10^−4^ ① +	10^−4^ ② +	10^−4^ ③ −	10^−5^ ① +	10^−5^ ② −
14	10^−1^ ① +	10^−2^ ② +	10^−3^ ① +	10^−3^ ② −	10^−3^ ③ −	10^−4^ ① +	10^−4^ ② +	10^−4^ ③ −	10^−5^ ① +	10^−5^ ② +
3	10^−1^ ① +	10^−2^ ② +	10^−3^ ① +	10^−3^ ② +	10^−3^ ③ +	10^−4^ ① +	10^−4^ ② +	10^−4^ ③ −	10^−5^ ① +	10^−5^ ② +
16	10^−2^ ① +	10^−2^ ② +	10^−3^ ① +	10^−3^ ② −	10^−3^ ③ +	10^−4^ ① +	10^−4^ ② +	10^−4^ ③ +	10^−5^ ① +	10^−5^ ② −
10	10^−1^ ① +	10^−2^ ② +	10^−3^ ① +	10^−3^ ② −	10^−3^ ③ +	10^−4^ ① +	10^−4^ ② +	10^−4^ ③ −	10^−5^ ① +	10^−5^ ② −
18	10^−2^ ① +	10^−2^ ② +	10^−3^ ① +	10^−3^ ② +	10^−3^ ③ −	10^−4^ ① +	10^−4^ ② +	10^−4^ ③ +	10^−5^ ① +	10^−5^ ② −
20	10^−1^ ① +	10^−2^ ② +	10^−3^ ① +	10^−3^ ② +	10^−3^ ③ +	10^−4^ ① +	10^−4^ ② −	10^−4^ ③ −	10^−5^ ① +	10^−5^ ② −
A2	10^−1^ ① +	10^−2^ ② +	10^−3^ ① +	10^−3^ ② +	10^−3^ ③ +	10^−4^ ① +	10^−4^ ② −	10^−4^ ③ −	10^−5^ ① −	10^−5^ ② −
A1	10^−1^ ① +	10^−2^ ② +	10^−3^ ① +	10^−3^ ② +	10^−3^ ③ −	10^−4^ ① +	10^−4^ ② +	10^−4^ ③ +	10^−5^ ① −	10^−5^ ② −
4	10^−1^ ① +	10^−2^ ② +	10^−3^ ① +	10^−3^ ② −	10^−3^ ③ −	10^−4^ ① +	10^−4^ ② −	10^−4^ ③ −	10^−5^ ① +	10^−5^ ② −

“+”: turbid enrichment culture tube; “−”: A negative enrichment culture tube that is not turbid or has precipitates and filamentous substances.; “Coarse font”: positive enrichment culture tube; “①, ②, ③”: Different culture tubes of the same dilution gradient.

**Table 4:** Intuitive analysis table.

Experimental Number	Chitosan (A)	L-rhamnose (B)	Sialic acid (C)	Galactose (D)	OD_600nm_
1	1	1	1	1	1.06
2	1	2	2	2	0.96
3	1	3	3	3	0.64
4	2	1	2	3	0.81
5	2	2	3	1	0.73
6	2	3	1	2	0.73
7	3	1	3	2	0.77
8	3	2	1	3	0.56
9	3	3	2	1	0.69
K_1_	2.66	2.64	2.35	2.48	
K_2_	2.27	2.25	2.46	2.46	
K_3_	2.02	2.06	2.14	2.01	
k_1_	0.89	0.88	0.78	0.83	
k_2_	0.76	0.75	0.82	0.82	
k_3_	0.67	0.69	0.71	0.67	
R	0.21	0.19	0.11	0.16	
Excellent level	A_1_B_1_C_2_D_1_				
Order	A > B > D > C				

## Data Availability

The original contributions presented in the study are included in the article, further inquiries can be directed to the corresponding author.
